# Can Proliferation Biomarkers Reliably Predict Recurrence in World Health Organization 2003 Defined Endometrial Stromal Sarcoma, Low Grade?

**DOI:** 10.1371/journal.pone.0075899

**Published:** 2013-10-11

**Authors:** Weiwei Feng, Anais Malpica, Ivar Skaland, Einar Gudlaugsson, Stanley J. Robboy, Ingvild Dalen, Keqin Hua, Xianrong Zhou, Jan P. A. Baak

**Affiliations:** 1 Department of Gynecology and Shanghai Key Laboratory of Female Reproductive Endocrine-Related Diseases, Obstetrics and Gynecology Hospital of Fudan University, Shanghai, China; 2 Department of Pathology, Obstetrics and Gynecology Hospital of Fudan University, Shanghai, China; 3 Departments of Pathology and Gynecologic Oncology, The University of Texas M.D. Anderson Cancer Center, Houston, Texas, United States of America; 4 Department of Pathology, Stavanger University Hospital, Stavanger, Norway; 5 Department of Research, Stavanger University Hospital, Stavanger, Norway; 6 Clinical Institute, Medical-Odontologic Faculty, University of Bergen, Bergen, Norway; 7 Departments of Pathology and Obstetrics and Gynecology, Duke University Medical Center, Durham, North Carolina, United States of America; Health Canada and University of Ottawa, Canada

## Abstract

An estimated 1500–3000 invasive Endometrial Stromal Sarcomas (ESS) cases annually occur worldwide. Before 2003, ESS was divided as low and high grade ESS based on mitotic activity. In 2003 the WHO changed the names, excluded mitoses and made nuclear atypia and necrosis the essential diagnostic criteria to distinguish ESS, Low Grade (ESS-LG, recurrence-free survival >90%) and Undifferentiated Endometrial Sarcoma (UES, poor prognosis). We have evaluated in WHO2003 defined ESS-LG whether proliferation biomarkers predict recurrence. Using survival analysis, the prognostic value of classical mitosis counts (Mitotic Activity Index, MAI) in haematoxyllin-eosin (H&E) sections, and immunohistochemical proliferation biomarkers (Ki-67 and PhosphoHistone-3 (PPH3)) were examined in 24 invasive endometrial stromal sarcomas. Three of 24 (12.5%) ESS-LG recurred. The MAI, PPH3 and Ki-67 were all prognostic (P = 0.001, 0.002 and 0.03). MAI values were >3 in the recurrent cases, but never exceeded 10 (the classical threshold for low and high grade). Non-recurrent cases had 0≤MAI≤3. PPH3 and Ki67 counts can be easier to perform than MAI and therefore helpful in the diagnosis of ESS, Low Grade. In conclusion, in this small study of WHO2003 defined ESS-LG, high levels of proliferation as measured by MAI, PPH3 and Ki-67 are predictive of recurrence. Larger studies are required to confirm these results.

## Introduction

Invasive endometrial stromal neoplasms are rare uterine tumors, accounting for 0.2 to 0.7% of all uterine malignancies and 15% of all uterine sarcomas [1], [2]. In Scandinavia, the incidence rate was 0.3/100,000 during 1978–2007 [3]. On the basis of these data, it can be roughly estimated that 1500–3000 invasive Endometrial Stromal Sarcomas (ESS) cases annually occur worldwide.

During the past decade, the rules for what constitutes an Endometrial Stromal Sarcoma (ESS) and what not have changed. Before 2003, invasive endometrial stroma tumors were classified according to their mitotic count per 10 high power fields (HPF) as low (<10 per 10 HPF) or high grade (≥10 per 10 HPF) [4]. In 2003, the World Health Organization (WHO) changed both the criteria used for the classification and the definition. First, mitosis counts are not used any more. Secondly, cellular atypia and tumor necrosis are the classifying diagnostic features. These two features distinguish Endometrial Stroma Sarcoma, Low Grade (ESS-LG), and Undifferentiated Endometrial Sarcoma (UES).

A number of tumors, whose mitosis counts were more than 10 but without nuclear atypia and necrosis, previously classified as high grade ESS, are now ESS-LG. On the other hand, some previously low grade ESSs are now UES. Because of changes in definition, studies on biologic and prognostic features before 2003 might have biases. Moreover, many studies since 2003 have still used the old classification. Thus, results of prognostic features in ESS-LG need to be thoroughly interpreted.

Due to the rarity of ESS-LG and UES, therapies used vary widely from observation without additional treatment after different surgery, to hormone therapy, chemotherapy and radiation therapy, either alone or in varying combinations [5]–[9]. A biomarker tailored personalized therapy may be useful for proper treatment.

Regarding the etiology, several studies suggested that ESS-LG and UES have distinct cytogenetic profiles. Two zinc finger genes, JAZF1 and JJAZ1, at the sites of the 7p15 and 17q21 breakpoints were the first translocations identified in endometrial stromal nodules and low grade ESS. The presence of JAZF1-JJAZ1 markedly inhibited apoptosis and induced proliferation rates [10]. Subsequently, rearrangements of JAZF1, SUZ12, PHF1 and EPC1 have been reported in endometrial stromal nodules (ESNs), ESS, and rarely in UES. The presence of detectable gene rearrangements in uterine ESS may predict better patient outcome [11]. In contrast to classic low grade ESS with JAZF1-SUZ12 fusions, YWHAE-FAM22 ESS displays high-grade histologic features and is associated with more aggressive disease course [12], [13]. As most pathology laboratories currently do not have access to translocation analysis, and FISH probes for the assessment of *JAZF1/JJAZ1* and other fusions are not commercially available, it is important to have easy and widely available methods allowing pathologists to assess which patients with an ESS are at high recurrence risk.

While WHO2003 ESS-LG tumors in general have a good prognosis and behave in a relatively indolent manner, late recurrences and distal metastases do occasionally occur. This has in recent years fostered a rebirth of studies of nuclear proliferation markers. The proliferation biomarker Cyclin D1 can be used as a simple immuno-histochemical surrogate biomarker for YWHAE-FAM22 ESS [14]–[16]. β-catenin [16], [17], p53 [18], [19] and p16 [20] also are mostly expressed in UES and associated with aggressive behavior. Another popular proliferation biomarkers is Ki-67 which in one study was expressed in 2 of 11 LGESS and predicted recurrence [21]. In agreement with this, Ki-67 and P53 expression occurred in 54% and 10% of LGESS (total 39 cases) and this also was associated with worse survival [22]. Although the pre-2003 diagnostic criteria were used in the latter two studies, it might be that increased proliferation in rigorously WHO2003 defined ESS, Low Grade tumors plays a prognostic role.

To further study this hypothesis, we have investigated WHO2003 defined ESS-LG with reasonably long term follow-up (median: 53, range: 24–83) [23] to identify features that are independently prognostic. The present study examines the value and limitations of nuclear proliferation markers, including mitoses counted in traditional haematoxyllin and eosin (H&E) stained microscopic sections, Ki-67 (sometimes called MIB-1 after the antibody for the staining of Ki-67 in paraffin sections) [21], [24] and a newer antibody that targets a nuclear antigen called PhosPhoHistone-3 (PPH3).

## Materials and Methods

This study was approved by the Institutional Review Board of the Obstetrics and Gynecology Hospital of Fudan University (FUOGH), Shanghai, China, where the patients were diagnosed and treated, and more recently by Regional Ethics Committee of Norway (REK-Vest, Bergen, Norway). Details for the patients have been described elsewhere [23], [25]. Medical records and microscopic sections of all tumors diagnosed as low and high grade endometrial stromal sarcoma (old classification) between 1992 and 2007 were retrieved from the FUOGH Gynecology and Pathology Department files. Stage was determined according to the 2009 International Federation of Gynecology and Obstetrics (FIGO) system for endometrial stromal tumors [26]. The cases were independently reviewed by experienced gynecological pathologists (JB, XZ) and only acceptable as ESS using the WHO2003 criteria [1] further refined by us as defined before, in order to remove from the WHO2003 definition definitional ambiguities of nuclear atypia and tumor necrosis [25]. We originally had 68 ESS-LG patients, but in 36 cases we only had H&E section (consultation slides and blocks had been submitted when the patient was referred to our hospital for treatment, but paraffin blocks had been returned to the original hospital). Of the 32 other cases, the fixed material in the paraffin blocks was too small or of poor quality in 8 cases. This left 24 ESS-LG good condition paraffin blocks adequate for immuno-histochemical studies. These 24 patients did not differ in any of the clinico-pathological features studied (P>0.10) from the original 68 ESS-LG patients.

### Tissues and studies based on haematoxyllin and eosin (H&E) stained sections

Mitotic activity index (MAI) was assessed in H&E stained 4 µm thick paraffin sections. Following Good Laboratory Practice criteria, the Standard Operating Procedure for the assessment of the MAI was the same as described in details elsewhere for breast cancer [27]. MAI assessment requires the count of all unambiguous mitotic figures per 10 high power fields (10 HPF), using a round microscopic field diameter of 450 micrometer or 1.59 mm2 total section area for 10 fields of vision at specimen level. The counts were obtained by different pathologists, including one of us who has had many years of experience in assessing mitotic counts (JB, who was blinded to the results of the routinely assessed MAI, and also to the original diagnosis, treatment and outcome). Where there were discrepancies of more than 2 mitoses with the original or each other's MAI assessment, we (JB, EG, XZ) re-assessed the case with a multi-headed microscope. Agreement was obtained in all cases.

### Immunohistochemistry for proliferation markers

Immunohistochemistry (IHC), antigen retrieval and antibody dilution were optimized prior to the study onset. To ensure uniformity, all sections were processed simultaneously. Four micrometer paraffin sections adjacent to the H&E sections used for histologic assessment were mounted onto Superfrost Plus slides (Menzel, Braunschweig, Germany), for PPH3 dried overnight at 60°C and for Ki-67 dried for one hour. Sections were deparaffinized in xylene and rehydrated in decreasing concentrations of alcohol. The Ki-67 and PPH3 antibodies have been described in detail before [28].The slides were dehydrated and mounted. All IHC staining procedures were performed using automated equipment. Ki-67 and PPH3 positive nuclei were independently counted by two of us (JB, EG) in the same measurement area as described above for the MAI (in 10 High Power Fields (HPFs) of vision (1.59 mm2). Ki-67 and PPH3 expressions were defined as the total number of positive nuclei and mitoses in 10 HPFs.

### Statistical Analysis

SPSS version 18 (SPSS; Chicago, Illinois, USA) was used for the statistical analyses. For evaluating the variables' prognostic significance, Kaplan-Meier survival analysis was performed. Recurrence free survival and overall disease related survival were endpoints (as the results for the two endpoints were essentially identical, only the recurrence-free survival results are presented). For age, MAI, Ki-67 and PPH3 which are continuous variables, the optimal prognostic threshold of sensitivity and specificity were detected by Receiver Operating Curve (ROC) analysis using the MedCalc® program (MedCalc, Mariekerke, Belgium).

## Results

The clinico-pathologic features for the subset of 24 patients for whom the tissue blocks were suitable for the current study were not statistically different from that of the full cohort of 68 ESS-LG patients presented elsewhere [25] (age, P = 0.65; frequency of ovary preserving surgical therapies, P = 0.41; mitotic activity index, P = 0.57; recurrence rates, P = 0.84). On this basis, the 24 ESS-LG cases in the current study were representative of the entire cohort of ESS-LG patients. Median age was 42 (range: 19–51) years. Table 1 shows the important clinico-pathologic and proliferation data.

**Table 1:** Clinicopathologic data from the patients.

	Recurrence/Number(%)	P value1)
Stage		
1	2/17 (11.7%)	
2	0/6 (0%)	
3	1/1 (100%)	0.21
MAI		
0–3	0/20 (0%)	
≥4	3/4 (75%)	0.001
PPH3		
0–21	0/19 (0%)	
>21	3/5 (60%)	0.002
Ki-67		
0–50	0/14 (0%)	
>50	3/10 (30%)	0.03

1) Kaplan-Meier survival analysis.

Median follow-up time for the 24 patients was 53 (range: 23–84) months. Three (12.5%) patients developed recurrences at 4, 23 and 35 months. The mean age of patients without and with recurrence was not different (41 and 43 years, P = 0.82). 17 of the tumors were stage 1, 6 were stage 2, and 1 was stage 3.Two patients with stage 1 and one with stage 3 disease recurred and stage was prognostically not significant.

**Figure 1:**
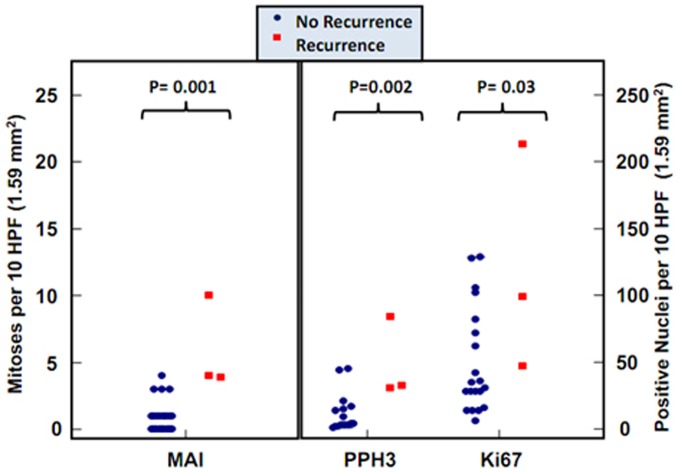
Dot plots of the MAI, Ki-67 and PPH3 in non-recurrent and recurrent ESS cases.

**Figure 2:**
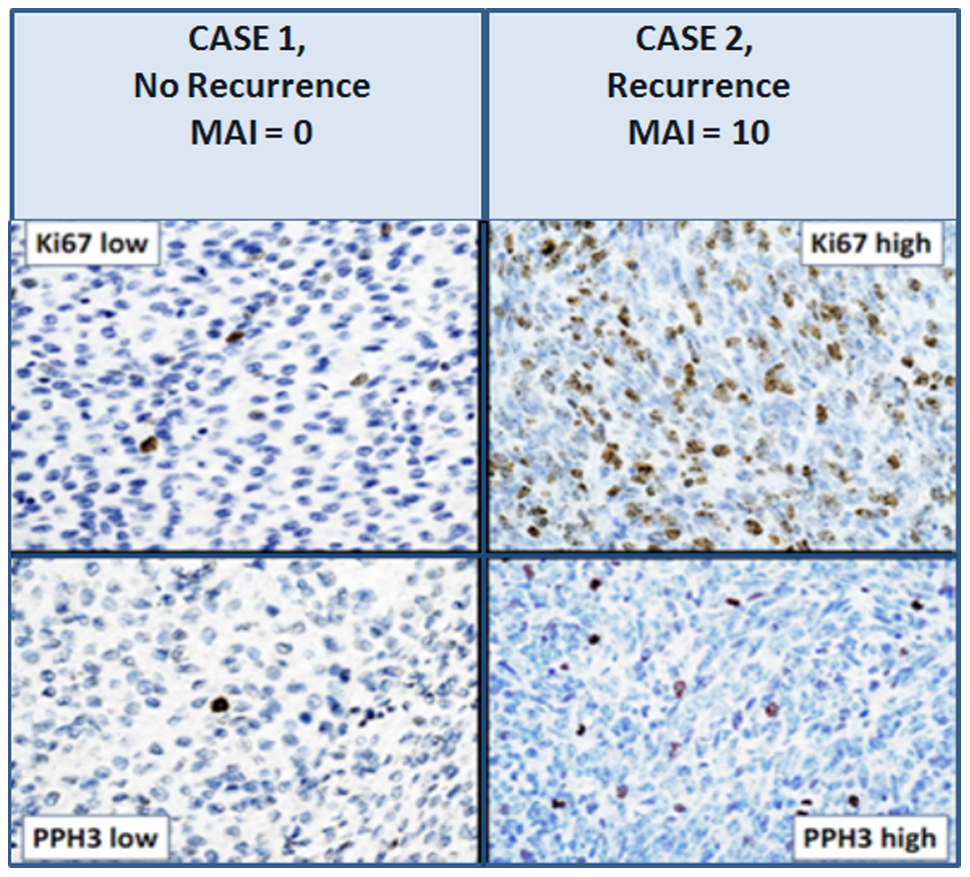
Examples of Endometrial Stroma Sarcomas showing parallel Ki-67 and PPH3 expression. A, B: Low Ki-67 and PPH3 expression in the same case. C, D: High expression of Ki-67 and PPH3 in the another case.

Like for the full cohort of 68 patients, the variable in the current cohort of 24 patients that correlated strongest with recurrence was the Mitotic Activity Index with a threshold of 0–3 versus 4 or more per 10 HPF (these 10 HPF represent 1.59 mm2 at specimen level) (P = 0.001), PPH3 (P = 0.002) and Ki-67 (P = 0.03) showed comparable results and correlated with recurrence, as is also clear from Figure 1. Not unexpectedly, the MAI was low (median 1/10 HPF) in most cases. Only 4 cases had a MAI of 4 or greater (Figure 1). Three of these had a MAI of 4, two recurred and one did not. The single case with a MAI of 10 recurred and died in spite of active salvage surgical, cytostatic and radiotherapy. The findings for PPH3 and Ki67 paralleled that of the MAI (Figure 1). Examples of paired Ki-67 and PPH3 in two cases with low and high MAI proliferation show that the expression patterns for Ki-67 and PPH3 are comparable (Figure 2).

## Discussion

Before 2003, Endometrial Stromal Sarcomas were traditionally divided as low and high grade, based on the mitotic counts (<10 versus ≥10 per 10 High Power Fields). The change by the WHO2003 of the definition and classification and nomenclature of “low and high grade ESS” into ESS, Low Grade and UES has a long history, going back to Evans in 1982 [29]. Moreover, around that time it was also shown that mitotic counts between different pathologists are not always well reproducible, due to the quality of the sections, differences in the inclusion criteria amongst pathologists of “*a mitotic figure*”, how and where to select fields of vision for mitosis counts and the wide variation of the area at specimen level of “10 high power fields of vision”. In spite of these influencing factors, mitoses can be well reproducible as was shown in the in the nationwide Dutch MMMCP protocol comparing mitosis counts in nearly 3500 consecutive breast cancers in 34 different pathology laboratories over a three years period. In that study the pathologists all used the same protocol and microscopic objective, resulting in a total measurement area of 1.59 mm^2^ at specimen level. However, other studies stating to have used “10 HPFs” have used objectives with a much larger diameter, resulting in a total measurement area of nearly 2.8 mm^2^ [2]. Such problems do not exist with the two essential diagnostic WHO2003 characteristics, the degree of atypia and extent of necrosis. The advantage of these criteria compared to mitosis counts and the fact that the classification is well reproducible and strongly prognostic [23] makes clear that the WHO2003 classification can be an improvement.

Nevertheless, about 10% of the WHO2003 ESS-LG still recurs and it would be of clinical significance to identify which cases do and which do not recur. Aided with the exact protocol available for assessment of Mitotic Activity Index and the immunohistochemical proliferation biomarkers Ki-67 and PPH3, a “diagnostic proliferation add-on” consisting of the MAI, Ki-67 and PPH3 could be of diagnostic value.

Due to the rarity of ESS-LG and UES, the number of studies since 2003 on ESS-LG and UES is limited [25] (see Table 1 in reference [25]). Moreover, some of these still use the old classification as low and high grade ESS, whereas others state that the WHO2003 has been used but the survival rates of ESS-LG are below 70%. This disagrees with the WHO2003 definition of ESS-LG cases which in general have an indolent behavior. As the women with the diagnosis of ESS-LG are relatively young (42 years), an accurate prediction of recurrence-or-not is of the utmost importance, as “high risk” ESS-LG could be considered more frequent follow-up.

The purpose of this study therefore was to identify whether proliferation biomarkers of Endometrial Stromal Sarcomas, Low Grade can help to predict recurrence. All three proliferation biomarkers (MAI, PPH3 and Ki-67) significantly predicted recurrence, thereby strengthening the historically known fact (from before the WHO2003 definition) that high proliferation is associated with an increased risk on recurrence.

The fact that all three proliferation features are prognostic is interesting as they represent different parts of the cell cycle and their expressions are only partly overlapping. This greatly strengthens the findings and underlines that their increased values in recurrent ESS-LG indeed reflect biological increased growth speed in the recurrent cases. It makes it also very unlikely that the increased proliferation detected is due to chance. Cells in their resting phase (G0-phase) show no activity for any of the three proliferation markers. Cells in the cell-division cycle or “cycling cells” are the cells preparing to divide. These cells all go through a sequence of identical phases (the so-called G1-, S-, G2 and M-phase) before they divide to form two identical daughter cells. The duration for the entire cell-division cycle process to go to completion varies greatly from one tissue to another, but generally takes 18–24 hours (sometimes shorter or longer). The M(itosis) phase, well known to pathologists as metaphases are visible during this phase, varies greatly but roughly accounts for approximately 4% of the cell cycle duration and typically is confined to the last hour of the cycle. Nearly all phases of cycling cells (G1-, S-, G2-) express Ki-67. The PPH3 antigen is expressed nearly exclusively in the cells late G2-phase (the nuclei of these cells already show coarse chromatin, just before becoming a mitosis) and M-phase (mitotic figures). Therefore, the frequency of PPH3 and Ki-67 positive cells understandably is higher than that seen for the MAI. Yet, all three proliferation markers tell the same story: increased proliferation in ESS-LG is associated with a significantly increased chance on recurrence.

In the past, the prognostic threshold for low and high grade ESS was 10, but we found that 4 or more mitoses per 10 HPF (with a total area scanned of 1.59 mm2 at specimen level) were significant in ESS-LG. This is very similar to the one found by Abeler et al [2] (after considering that she used a nearly 2× larger measurement area) and Ashraf [30] who had a threshold of 5, suggestively close to ours.

The cell proliferation index assessed by using MIB-1 antibody against the Ki-67 antigen is widely used and accepted because of its crisp contrast-rich staining pattern and being sympathetic to less than stellar managing of tissue acquisition and tissue handling. Many studies have found it to be diagnostically and prognostically useful and in a small series of 11 low grade ESS it successfully predicted the 2 patients who developed recurrent disease [21]. PPH3 also gives excellent staining results. In short, simultaneous assessment of MAI, Ki-67 and PPH3 therefore may be considered in case of ESS-LG, to identify the about 10% of cases with an increased risk on recurrence. To obtain reliable results for the MAI, strict adherence to the protocol we have previously described is of the utmost importance [28].

Other studies also found a relation between high-risk on recurrence and increased proliferation in ESS-LG [31]. Moreover, ESSs expressed MIB-1 significantly more frequently than ESNs [32].The role of EGFR is less clear. Up to 70% of low-grade endometrial stromal sarcomas showed positive reactions for EGFR [33] which led to the interesting suggestion that this may provide the basis for a new therapeutic strategy using monoclonal antibodies against EGFR. However, others found a much lower expression (11%) and amplification of EGFR gene was not found at all. Results on EGFR overexpression without amplification confirmation therefore should be interpreted with caution [34],[35].

Of course, the therapeutic consequences of increased recurrence rate in ESS-LG with elevated proliferation are a matter of further studies. Li [36] found that surgeries preserving ovarian function increased the risk of recurrence compared with those surgeries sparing ovarian function, similar to our findings [25]. This is biologically quite understandable in view of the young age of the patients, the high levels of estrogens and estrogen receptors of ESS-LG. On the other hand, we did not find that extensive radical operations, lymphadenectomy, omentectomy, adjuvant chemotherapy and radiotherapy in general improve prognosis in ESS-LG [37], but this may be different for ESS-LG with increased proliferation. Considering that the responsiveness to tyrosine kinase inhibitors (TKR) is known to be related to the presence of specific activating mutations or gene over-expression, which are not detectable in ESS, TKR immunohistochemical over-expression alone cannot be considered as a reliable marker for targeted therapies in ESS [38].

In spite of these promising and biologically understandable results, the number of cases was small (n = 24, with one case being FIGO stage3). On the other hand, the differences were highly significant (P<0.0001) in 2 of the 3 markers and the three different proliferation biomarkers all point into the same direction. The likelihood that the differences are due to chance, when there are no real differences, are therefore very low. Based on the national incidence rates of ESS in the whole of Norway, with less than 90 cases of ESS and UES in more than 30 years, the chances to soon get a large enough series of WHO2003defined ESS-LG, with long enough follow-up and rather homogeneous treatment, are slim. We call for international multicenter collaborative studies to validate the current results. The quality of the immuno-histochemical stainings should be carefully controlled, preferably by means of an external international Quality Control and Assurance system, such as NordiCQ [39].

In conclusion, in FIGO2009, WHO2003 defined ESS-LG, elevated levels of proliferation as measured by MAI, PPH3 and Ki-67 seem predictive of tumors that will recur. Larger independent studies are required to confirm these results.
